# Transport in a Two-Channel Nanotransistor Device with Lateral Resonant Tunneling

**DOI:** 10.3390/mi15101270

**Published:** 2024-10-19

**Authors:** Ulrich Wulf, Amanda Teodora Preda, George Alexandru Nemnes

**Affiliations:** 1 Faculty 1, Brandenburg University of Technology Cottbus-Senftenberg, Platz der Deutschen Einheit 1, Konrad-Wachsmann-Allee 13, 03046 Cottbus, Germany; 2 Faculty of Physics, University of Bucharest, Atomistilor 405, 077125 Magurele-Ilfov, Romania; amanda.preda@nipne.ro (A.T.P.); alexandru.nemnes@nipne.ro (G.A.N.); 3 Horia Hulubei National Institute for Physics and Nuclear Engineering, Reactorului 30, 077125 Magurele-Ilfov, Romania

**Keywords:** lateral resonant tunneling, two-channel transistor, nanotransistor, quantum transport, Landauer–Büttiker formalism, R-matrix method

## Abstract

We study field effect nanotransistor devices in the Si/SiO_2_ material system which are based on lateral resonant tunneling between two parallel conduction channels. After introducing a simple piecewise linear potential model, we calculate the quantum transport properties in the R-matrix approach. In the transfer characteristics, we find a narrow resonant tunneling peak around zero control voltage. Such a narrow resonant tunneling peak allows one to switch the drain current with small control voltages, thus opening the way to low-energy applications. In contrast to similar double electron layer tunneling transistors that have been studied previously in III-V material systems with much larger channel lengths, the resonant tunneling peak in the drain current is found to persist at room temperature. We employ the R-matrix method in an effective approximation for planar systems and compare the analytical results with full numerical calculations. This provides a basic understanding of the inner processes pertaining to lateral tunneling transport.

## 1. Introduction

In this study, we consider field effect nanotransistor devices based on lateral resonant tunneling between two parallel conduction channels: One channel is selectively coupled to the source contact and the other channel is selectively coupled to the drain contact. A source-drain current thus can only flow when there exists tunneling transport between the two channels. Because of strong level quantization in each channel, tunneling only occurs under special resonance conditions which can be controlled with very small voltages applied to two control gates. This opens the way to low operating voltages around 100 mV suitable for low energy applications.

A similar resonant tunneling mechanism was employed in the 1990s in double electron layer tunneling transistors (DELTTs) in the GaAs/AlGaAs material system. Here, two selectively coupled electron layers in GaAs are separated by an AlGaAs tunneling barrier. Experimental solutions to this central technological problem are demonstrated in Refs. [1,2,3,4,5,6,7,8,9,10,11,12,13,14]. In a DELTT, both lateral dimensions of the electron layers lie typically in the range of tens of micrometers while the widths of the electron layers and the tunneling barrier in the perpendicular direction lie in the range of tens of nanometers. The electron layers thus assume the character of a two-dimensional (2D) electron gas which is loaded from dopants in layers above and below the electron layers and transport was discussed in terms of 2D–2D tunneling [5,15,16,17]. Under these conditions, resonant tunneling peaks were found in the transfer characteristics at low temperatures, typically in the Kelvin range.

**Figure 1:**
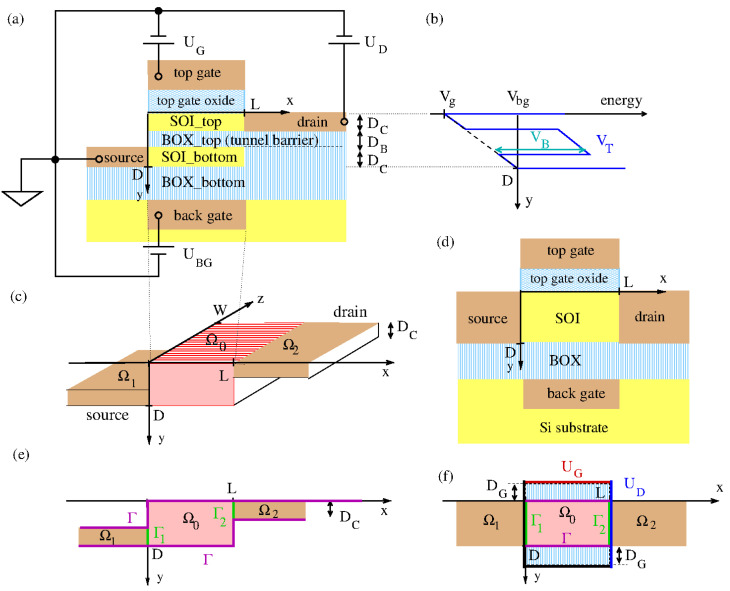
(**a**) Realization of the 2CTFET based on a double SOI-substrate as described in Ref. [18]. (**b**) Simple transverse model potential $V_{T}$ for a double quantum well as taken from Equation (3) for negative $V_{g}$ (positive $U_{G}$) and $V_{bg}=0$. (**c**) The structure blocks defined in [19] identified for the case of a 2CTFET: The source and the drain contact in the volumes $\Omega_{1}$ and $\Omega_{2}$. They are connected to the central scattering volume $\Omega_{0}$ (device kernel, red) via the surfaces $\Gamma_{1/2}$ (green). (**d**) Schematic representation of the structure elements of an SOI transistor. (**e**) For the Wigner–Eisenbud functions $\chi_{l}\left(\vec{r}\right)$, one has in the R-matrix formalism the boundary conditions $\chi_{l}\left(\vec{r}\in \Gamma \right)=0$ (Γ in magenta) and $\chi_{l;y}\left(\vec{r}\in \Gamma_{1}\right)=0$ and $\chi_{l;y}\left(\vec{r}\in \Gamma_{2}\right)=0$, where $\chi_{l;y}$ is the partial derivative in the y-direction. These boundary conditions are incompatible with the simplifying product ansatz $\chi_{l}\left(\vec{r}\in \Omega \right)=\chi_{\lambda }\left(x\right)\phi_{k}\left(y,z\right)$ as in Equation (37) of [19]. (**f**) Modified boundary conditions for the $\chi_{l}$ eigenfunctions compatible with the simplifying product ansatz which we assume for our calculations in the EAPS. The boundary conditions for the Poisson equation are depicted by thick solid lines, in black (0V), red ($U_{G}$) and blue ($U_{D}$) colors. The domain includes the top and back gate oxide regions.

With the substantial progress in semiconductor technology, it is now possible to fabricate double quantum wells in the technologically relevant Si/SiO_2_-material system in double silicon-on-insulator (SOI) substrates ([18], see Figure 1). Furthermore, it has become possible to shrink the device lengths in the transport direction to the size of tens of nanometers, i.e., by a factor of a thousand compared to the DELTTs. Transport is then described by coherent wave functions ranging from source to drain [19]. Because of the global coherence in the transport direction from source to drain, tunneling between the electron layers assumes the character of lateral resonant tunneling rather than 2D–2D tunneling. Because of this essential difference in the device operation we call the described Si-based devices two-channel tunneling FETs (2CTFETs).

We calculate quantum transport in a 2CTFET using the R-matrix approach developed in Refs. [19,20,21]. Our approach has been confirmed in applications to nano-MOSFETs [22] and SOI transistors [23]. For the R-matrix method, we first employ the effective approximation for planar systems (EAPS, see Appendix E) which provides a physical understanding of the basic inner processes pertaining to lateral resonant tunneling transport. We then compare with the results of fully numerical calculations (2D model). One can now identify a number of features of the 2CTFET which are favorable for applications: First, while MOSFETs rely on thermal activation, 2CTFETs work with resonant tunneling which is independent of the temperature. Second, in comparison with DELTTs, the conduction channels are loaded from the lateral source and drain where the chemical potential can be increased up to the regime of electron volts in the limit of heavy doping and narrow contacts (see Appendix B), much more than the thermal energy at room temperature of 25 meV. It is therefore conceivable that thermal effects play a minor role in the 2CTFET and, indeed, we see that the resonant tunneling peak in the transfer characteristic persists at room temperature. Moreover, the extremely efficient injection from the heavily doped narrow source/drain contacts and the much lesser scattering in the short, nearly ballistic conduction channels favor relatively high drain currents.

## 2. Model

**Figure 2:**
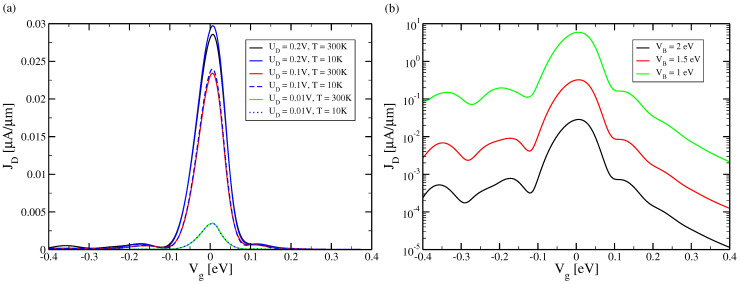
The drain current per width $J_{D}=I_{D}/W$ vs. $V_{g}$ for $D_{B}$ = 2 nm. (**a**) For $V_{B}$ = 2 eV: $U_{D}$ = 0.2 V, *T* = 300 K (black); $U_{D}$ = 0.2 V, *T* = 10 K (blue solid); $U_{D}$ = 0.1 V, *T* = 300 K (red); $U_{D}$ = 0.1 V, *T* = 10 K (blue dashed); $U_{D}$ = 0.01 V, *T* = 300 K (green); $U_{D}$ = 0.01 V, *T* = 10 K (blue dotted). (**b**) For $U_{D}$ = 0.2 V and *T* = 300 K: $V_{B}$ =2 eV (black), 1.5 eV (red) and 1 eV (green).

We consider the realization of a 2CTFET on a double SOI substrate which is shown schematically in Figure 1. The 2CTFET can be derived from an SOI transistor on a standard SOI wafer as follows: As is well known, in an SOI transistor one adds to the structure elements of a standard SOI wafer (Si substrate, buried oxide (BOX) and Si film) the further elements source, drain, back gate, top gate oxide and top gate (see Figure 1d). Then, in a double SOI substrate an extra buried oxide BOX_top is added to the standard SOI wafer (see Figure 1a, [18]). In consequence, the Si film is split into two separate layers, SOI_top and SOI_bottom, which can be as narrow as 2 nm. The two layers thus assume the character of quantum wells which are coupled by the buried oxide BOX_top acting as a tunneling barrier. In the shown 2CTFET, the quantum well in SOI_bottom is selectively contacted to the source and the quantum well in SOI_top is selectively contacted to the drain. In the strong barrier limit, a drain current from source to drain only occurs under lateral resonant tunneling conditions leading to the resonant tunneling peak in the transfer characteristics as shown in Figure 2.

**Figure A4:**
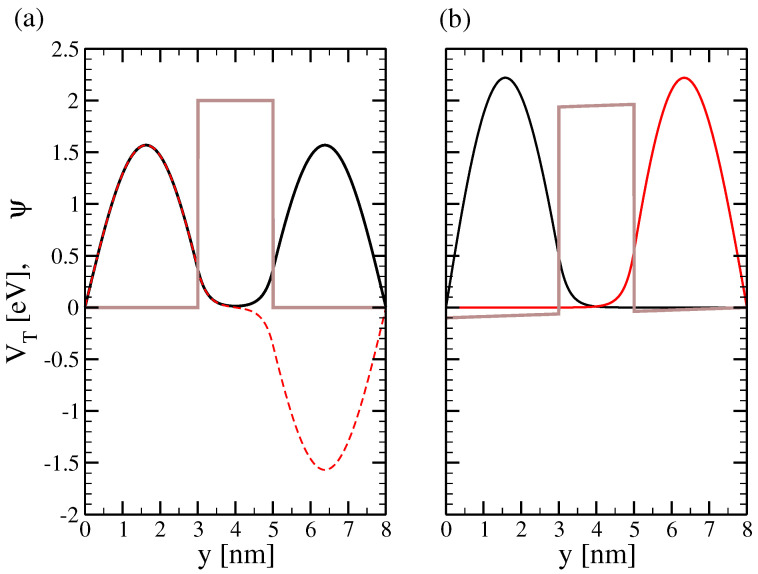
The two lowest transverse modes in the kernel for $V_{B}=2$ eV, $k_{y}=1$ (black) and $k_{y}=2$ (red). In brown color, the transverse potential $V_{T}\left(y\right)$. (**a**) $V_{g}=0$ (symmetrical case in resonance) and (**b**) $V_{g}=-0.1$ eV (asymmetrical case OFF resonance).

We consider a simple model potential $V(\vec{r})$ for the 2CTFET:

1. In the grounded source contact $\Omega_{1}$, we set $V(\vec{r}\in \Omega_{1})=0$
2. In the drain contact $\Omega_{2}$, we set $V\left(\vec{r}\in \Omega_{2}\right)=-eU_{D}$.
3. In the device kernel $\Omega_{0}$ (‘scattering area’), we cut off the wave functions in the top-gate oxide and the BOX_bottom. In the remaining tunneling coupled quantum wells, we choose a piecewise linear potential of the separable form
  (1) $$V\left(\vec{r}\in \Omega_{0}\right)=V_{T}\left(y\right)+V_{L}\left(x\right).$$
  In the transport direction (*x*-direction), there is a linear potential drop of the applied drain voltage $U_{D}$
  (2) $$V_{L}\left(x\right)=-\frac{x}{L}eU_{D},$$
  where *L* is the channel length. We write the transverse potential in the device kernel in the form
  (3) $$V_{T}\left(0\leq y\leq D\right)=V_{B}\Theta \left(y-D_{C}\right)\Theta \left(D_{C}+D_{B}-y\right)+V_{g}+\left(V_{bg}-V_{g}\right)\frac{y}{D}$$

(See Figure 1b). Here, $V_{B}$ and $D_{B}$ are the height and thickness of the tunnel barrier (BOX_top) along the y-direction. Furthermore, $D_{C}$ is the thickness of the two quantum wells (SOI_top and SOI_bottom) formed between the tunneling barrier and the top gate oxide and the BOX_bottom. Finally, $V_{g}=V_{T}\left(y=0\right)$ is the transverse potential on the upper edge of the silicon film and $V_{bg}=V_{T}\left(y=D\right)$ that on the lower edge. For $V_{g}=V_{bg}$, the transverse potential $V_{T}\left(y\right)$ is symmetrical and the quantum levels in the isolated quantum wells coincide. One obtains in the interacting system a series of pairs of resonantly interacting quantum wells. As described in detail in Appendix D, these two interacting quantum levels lead to two wave functions (one symmetrical and one anti-symmetrical) which are localized *at the same time* in both quantum wells, thus allowing the electron to interchange between the two quantum wells (see Figure A4a). At $V_{g}\neq V_{bg}$, out of resonance, this interaction is broken. The quantum wells become isolated and the current from source to drain becomes very small. The results are the narrow resonant tunneling peaks shown in Figure 2.

In our calculations, we use the parameter values listed in Table 1.

Inserting in the Schrödinger equation the *z*-independent potential $V\left(\vec{r}\right)=V\left(x,y\right)$, one obtains with the ansatz

(4) $$\Psi \left(\vec{r}\right)=\psi \left(x,y\right)\sqrt{\frac{2}{W}}\sin\left(\frac{n_{z}\pi }{W}z\right)$$

the two-dimensional Schrödinger equation

(5) $$\left[-\frac{\hbar^{2}}{2m^{*}}\left(\frac{\partial^{2}}{\partial x^{2}}+\frac{\partial^{2}}{\partial y^{2}}\right)+V\left(x,y\right)-E^{xy}\right)\psi \left(x,y\right)=0$$

with the energy of the motion in the x-y-direction $E^{xy}=E-\left(\hbar^{2}/2m^{*}\right){\left(n_{z}\pi /W\right)}^{2}$ where *E* is the total energy. For the calculation of I-V-traces of the 2CTFET, we use the Landauer–Büttiker formula for the drain current

(6) $$I_{D}=\frac{2e}{h}N_{V}\int_{0}^{\infty }dE^{xy}\left[S\left(E^{xy}-\mu \right)-S\left(E^{xy}-\mu +eU_{D}\right)\right]T^{2d}\left(E^{xy}\right)$$

with the valley degeneracy $N_{V}$ [19]. In the subsequent sections, we describe the further quantities in this expression in detail:

1. The chemical potential μ in the source in Appendix B
2. The supply function S(α) in Appendix C
3. For the evaluation of the two-dimensional current transmission $T^{2d}$, we apply in Section 3.1 the EAPS and in Section 3.2 the fully numerical 2D model.

**Figure A9:**
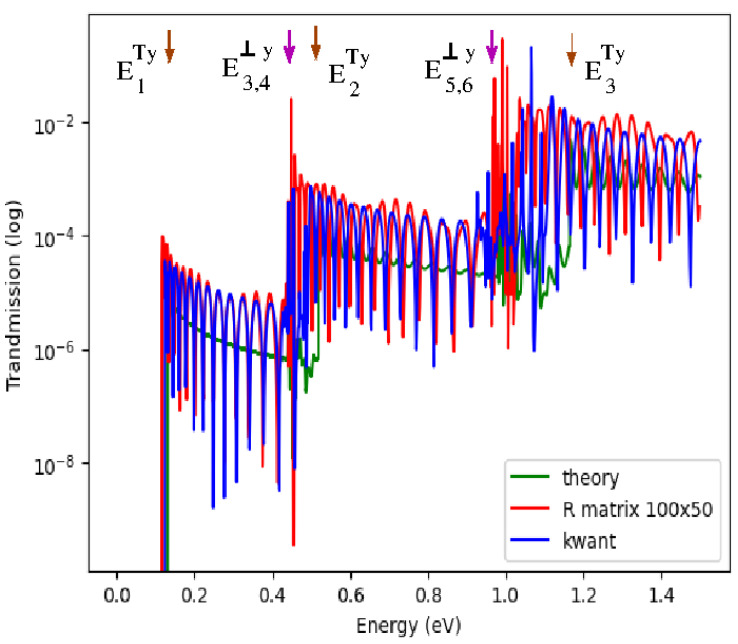
Comparison of the two-dimensional current transmission $T^{2d}$ in EAPS (green line) with fully numerical evaluations using the R-matrix method (red line) and Kwant (blue line). The arrows mark the energies of the tranverse modes.

Our 2D model is based on either the full R-matrix method [24] or Kwant [25]. The Kwant simulation tool is an open source Python library designed for transparent quantum transport simulations based on the tight binding formalism and a numerical method known as wave function matching (WFM) [26,27]. Wave function matching is a technique employed to compute the scattering matrix of a mesoscopic system that can also be described by a Hamiltonian in tight binding form and which is also suitable for *ab initio* calculations. In a continuous description, the method is based on slicing the Hamiltonian on the transport direction and imposing matching conditions for the wave function. The WFM is closely related to other methods, like the R-matrix method and Green’s function techniques. However, in the R-matrix method, the Hamiltonian of the central system is diagonalized only once, independent of the total energy and the matching conditions are imposed between the scattering region and the leads. On the other hand, Green’s function techniques are more general and one may also include dissipative effects. For non-interacting systems, these methods provide consistent results as is shown for R-matrix and WFM implemented in Kwant (see Figure A9).

The scattering potential is determined by solving the Poisson equation with boundary conditions set by $\left(U_{D},U_{G}\right)$, while the system is considered invariant in the *z*-direction (see Figure 1f). The Dirichlet type boundary conditions for the potential are set on a rectangular shaped region, defined by the intersection of the leads with the scattering region, $\Omega_{s}\cap \Omega_{0}$, with additional 8 nm buffers of oxide. The remaining segments are located at the positions of the top and back gates.

## 3. Results

### 3.1. Effective Approximation for Planar Systems (EAPS)

**Figure 3:**
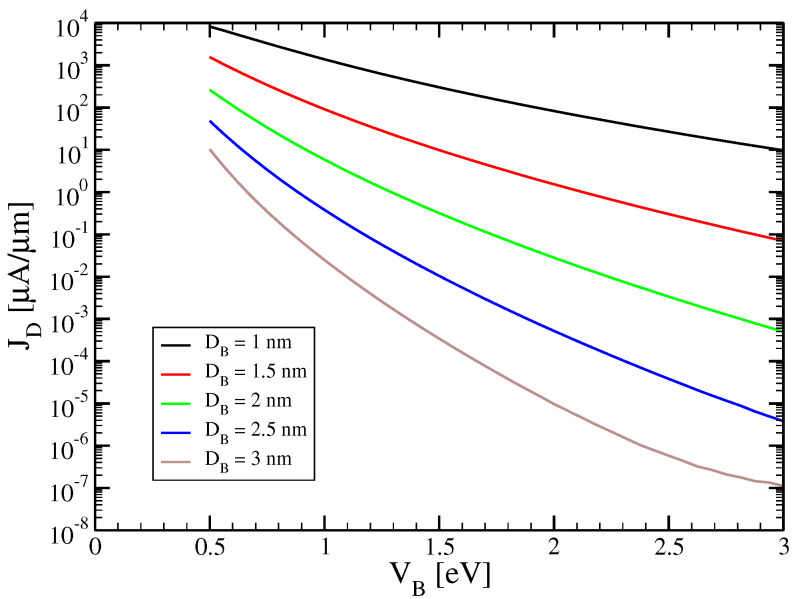
Maximum of the tunneling peak associated with Figure 2b taken at $V_{g}$ = 0 V vs. barrier height $V_{B}$ at drain voltage $U_{D}$ = 0.2 V. The barrier thickness is varied, taking the values $D_{B}$ = 1 nm (black), 1.5 nm (red), 2 nm (green), 2.5 nm (blue) and 3 nm (brown).

In the following plots, we evaluate in the limit W→∞ the quantity $J_{D}=I_{D}/W$ which is usually given in experimental work. Here, Equation (A10) is used for the supply function which is the only quantity depending on the width making sure that $I_{D}$ scales linearly with *W*. In Figure 2, we show the calculated transfer characteristics $J_{D}$ vs. $V_{g}$ of the 2CTFET. A central narrow resonant tunneling peak around zero control voltage $U_{G}=U_{BG}=0$ V is found with a small full width at half maximum of ∼100 mV. At higher control voltages, there are much smaller satellite peaks. As expected from the quantum mechanical nature of the resonant tunneling mechanism, the tunneling peak is nearly unchanged with varying device temperature, growing very weakly with decreasing temperature. This is opposite to what is found in a MOSFET. In Figure 3, it is demonstrated that the maximum of the tunnelling peak decreases strongly, nearly exponentially, with increasing barrier height and barrier thickness.

**Figure 4:**
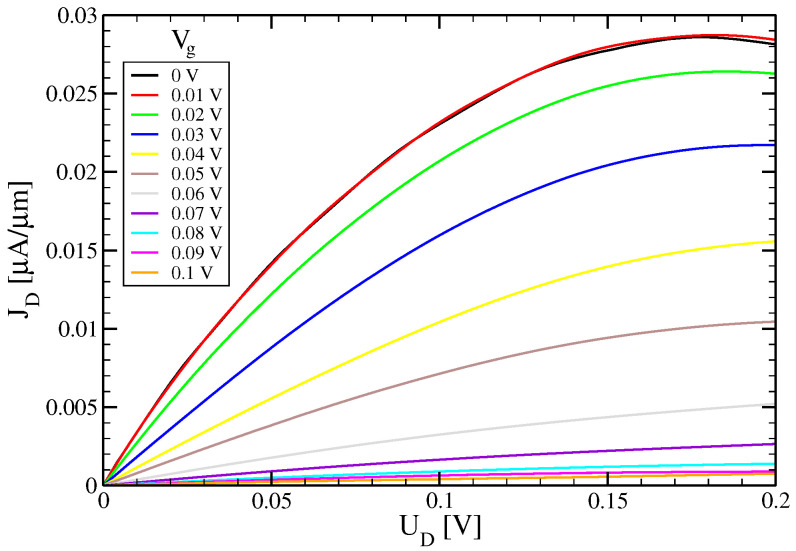
Output characteristic for $V_{B}$ = 2 eV, *T* = 300 k and $D_{B}$ = 2 nm: $V_{g}$ = 0 V (black), 0.01 V (red), 0.02 V (green), 0.03 V (blue), 0.04 V (yellow) 0.05 V (brown), 0.06 V (grey), 0.07 V (violet), 0.08 V (cyan), 0.09 V (magenta) and 0.1 V (orange).

In Figure 4, we plot the output characteristics of the 2CTFET. Since here we are interested in the low-voltage properties of the 2CTFET, we examine gate and drain voltages below 0.2 V as an operating voltage. The basic structure of the output characteristics resembles that of a MOSFET. However, as can be expected from the different transport mechanisms, the drain current of the 2CTFET becomes smaller with increasing gate voltage, opposite to the MOSFET. Also, there is no clear saturation voltage. Finally, the traces for small gate voltages develop at higher drain voltages a weak negative differential conductance. A negative differential conductance in the output characteristic is typical for resonant tunneling devices [28,29] and it is also present in DELTTs.

### 3.2. Full Numerical Calculations on 2D Models

**Figure 5:**
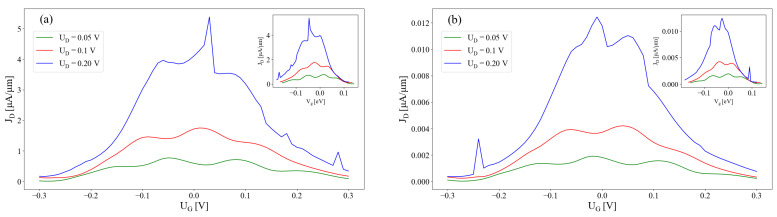
Drain current vs. gate voltage for several drain voltages, $U_{D}=0.05,0.1,0.2$ V, calculated using the tight-binding model, for (**a**) $V_{b}=1$ eV and (**b**) $V_{b}=2$ eV. The decrease in $J_{D}$ with $U_{G}$ is similar to the analytical calculation shown in Figure 2b. The inset shows the same data plotted against the potential energy, $V_{g}$, found midway (x=L/2) at the edge of the upper channel (y=0).

**Figure 6:**
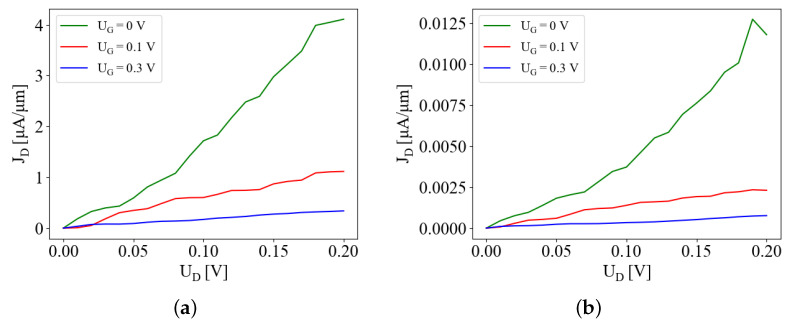
Drain current vs. drain voltage for several gate voltages, $U_{G}=0,0.1,0.3$ V, calculated using the tight-binding model, for (**a**) $V_{b}=1$ eV and (**b**) $V_{b}=2$ eV. An almost linear increase is observed at low $U_{D}$ voltages.

**Figure 7:**
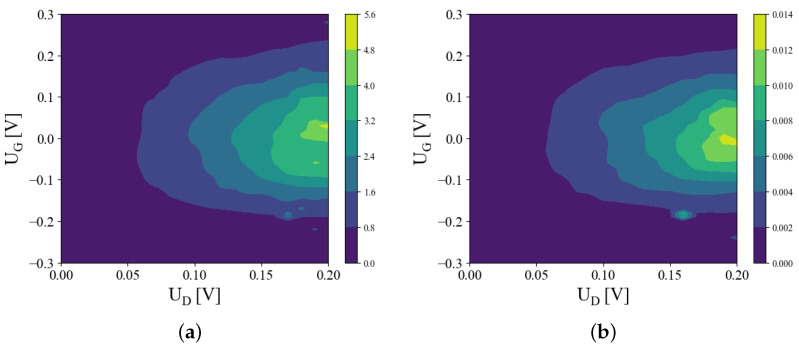
Heat maps of the drain current vs. $\left(U_{D},U_{G}\right)$ voltages, for (**a**) $V_{b}=1$ eV and (**b**) $V_{b}=2$ eV. A peak in $J_{D}$ is evidenced for large $U_{D}$ voltage and low values of $U_{G}$. The normally ON state of the 2CTFET can be switched to an OFF state by increasing $|U_{G}|$.

Figure 5 shows the behavior of the drain current as the gate voltage is varied, for several values of the drain bias. For $U_{G}=0$, the two quantum wells are symmetric and the electron wave functions are localized in both channels, leading to a significant current flow into the drain. In contrast, by increasing the magnitude of the gate voltage, $|U_{G}|$, the asymmetry of the two quantum wells becomes larger, resulting in localization of the wave functions in each of the two channels. In effect, the source-to-drain transport is diminished, similar to the analytical results show in Figure 2b. The normally ON state of the 2CTFET at $U_{G}=0$ V is thus turned into an OFF state. Unlike the analytic model, the solution domain of the Poisson equation included the top and bottom oxide layers (see Figure 1a), which in effect decrease the potential drop on the active region $\Omega_{0}$. For a proper comparison, we plotted in the insets of Figure 5 the drain currents against the potentials found at the upper edge of the drain channel (x=L/2, y=0, see Figure 1c). By varying the drain voltage, we observe an almost linear increase in the drain current, as depicted in Figure 6, for two barrier sizes ($V_{b}=1$ eV and $V_{b}=2$ eV) and several $U_{G}$ values. When $U_{G}$ is increasing, the slopes $dJ_{D}/dU_{d}$ are decreasing, which is consistent with the characteristics presented in Figure 4, reflecting the ON–OFF transition. For a more comprehensive overview on the dependence of J-V characteristics on the bias conditions, we plotted in Figure 7 contour plots of $J_{D}$ vs. $\left(U_{D},U_{G}\right)$ for $V_{b}=1$ eV and $V_{b}=2$ eV. This confirms the appearance of narrow maxima in the drain current around $\left(U_{G},U_{D}\right)=\left(0,0.2\right)$ V.

**Figure 8:**
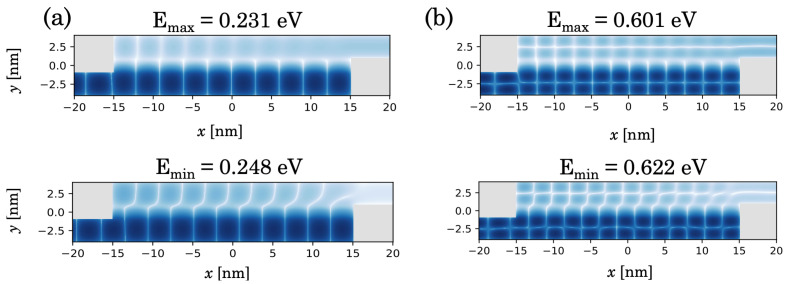
In-phase and out-of-phase matching of quasi-stationary wave functions (absolute value) in the two channels, obtained for $V_{b}=2$ eV: (**a**) energies E=0.231 eV and 0.248 eV; (**b**) energies E=0.601 eV and E=0.622 eV. These correspond to peaks and dips in the transmission function in Figure A9.

The transmission function shown in Figure A9 presents a series of sharp peaks which decrease in amplitude with total energy *E*, while their widths increase. For each transverse mode, a matching condition between the maxima and minima of the quasi-stationary waves in the two channels occurs for certain values of the total energy, leading to narrow peaks in the transmission. We call this in-phase wave function-matching condition and this is exemplified for E=0.231 eV in Figure 8a, for the first transverse mode. A dip in the transmission function corresponds to out-of-phase quasi-stationary waves in the two channels, as indicated, e.g., for E=0.248 eV. One obtains a similar picture for the second transverse mode, as one can see from Figure 8b. For the in-phase condition, the large overlap of the wave function favors the tunneling between the two channels, which is minimized for the out-of-phase condition. At the same time, from Figure A9 one can see that the oscillation period in the transmission function increases with the total energy. This can be explained by observing the overlap of the two stationary waves as the longitudinal k-vector is increased. Moreover, one should note that, as the electrons are incoming from the left in the lower channel, one expects in the upper channel a transition from quasi-stationary states to purely outgoing waves in the drain lead, which is visible near the interface with the drain.

Further investigations shall be focused on the effects of the Coulomb interaction. In confined quantum dot systems in SiO_*x*_ films, weakly coupled to the leads, Coulomb blockade is manifested [30]. Although the transistor system has a much stronger coupling between the leads and the active region, the Coulomb interaction can redefine the transversal mode energies, introducing energy shifts in the resonant transmission conditions.

## 4. Summary

We study the transport properties of field effect nanotransistor devices which are based on lateral resonant tunneling between two parallel conduction channels. In the transfer characteristics, we find narrow resonant tunneling peaks around zero control voltage allowing one to switch the drain current with small control voltages. This opens the way to low-energy applications. Furthermore, we study in an effective approximation to the general R-matrix approach the basic inner processes pertaining to lateral resonant tunneling transport. This analysis provides an in-depth understanding of the tunneling process, revealing the complex structure of the transmission function. Analytical calculations accurately provide the positioning of the transverse modes and the distribution of transmission peaks, which follows from the matching of quasi-stationary wave functions in the two channels. The consistency of the EAPS is further confirmed by numerical calculations on 2D models, using the R-matrix method and Kwant simulation package.

## Figures and Tables

**Table 1:** Notations and parameter values, above fixed values, below varied values.

Quantity	Notation	Values
Thickness of SOI_top and SOI_bottom	$D_{C}$	3 nm
Isotropic effective mass	$m^{*}$	$0.32\times m_{0}$
Valley degeneracy	$N_{V}$	6
Channel length	*L*	30 nm
Doping in source/drain contact	$N_{D}$	5 × 10^20^ cm^−3^
Potential at interface BOX_bottom/SOI_bottom	$V_{bg}$	0
Gate insulator thickness	$D_{G}$	8 nm
Width of the 2CTFET in the *z*-direction	*W*	up to *∞*
Height of tunnel barrier	$V_{B}$	1 eV or 2 eV
Potential at interface between top gate oxide/SOI_top	$V_{g}$	−0.2 eV ≤$V_{g}$≤ 0
Drain voltage	$U_{D}$	0 or 0.2 V
Device temperature	*T*	300 K or 10 K
Tunnel barrier thickness (BOX_top, SiO_2_)	$D_{B}$	1 nm to 3 nm, mainly 2 nm

**Figure A1:**
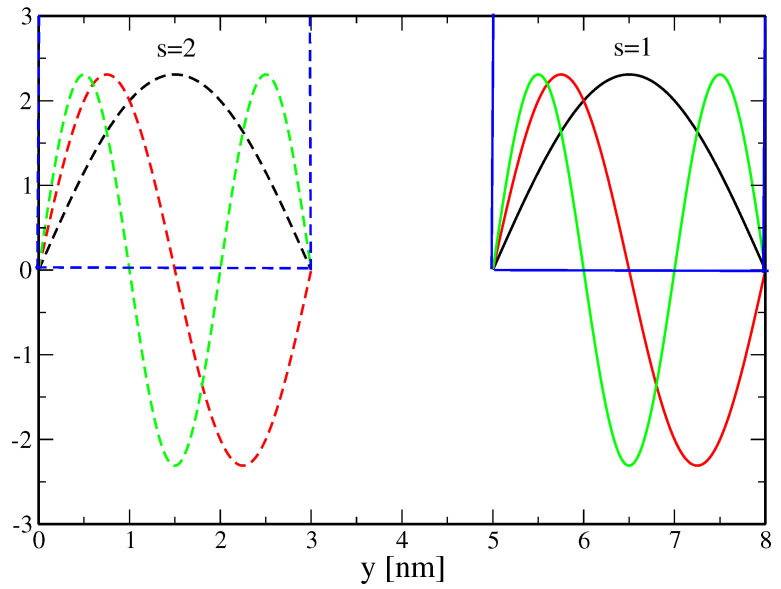
The transverse modes in the contacts $\Phi_{s,n_{y}}$ for s=1 (**solid lines**) and for s=2 (**dashed lines**): $n_{y}=1$ (**black**), $n_{y}=2$ (**red**) and $n_{y}=3$ (**green**). In blue, the confinement potential in the source (**solid line**) and in the drain (**dashed line**).

**Figure A2:**
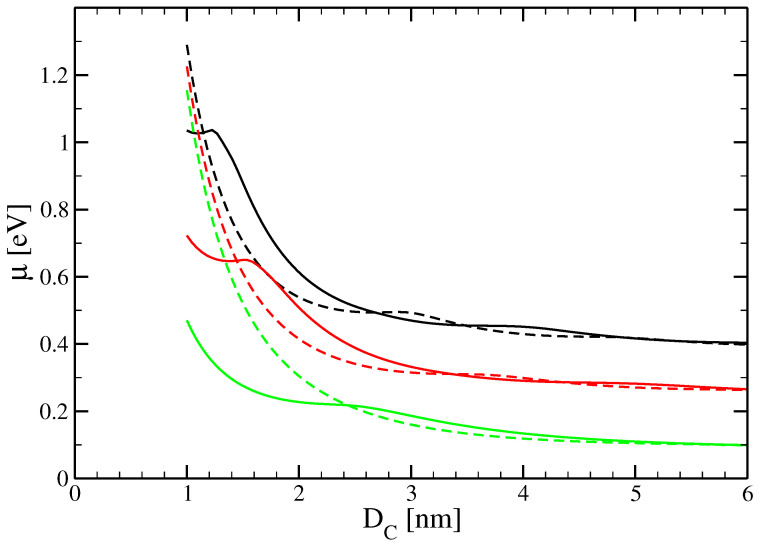
The chemical potential in the source versus depth of the source for $N_{D}=10^{21}$ cm^−3^ (**black**), $5\times 10^{20}$ cm^−3^ (**red**) and $10^{20}$ cm^−3^ (**green**). Solid lines for the six constant energy ellipsoids in silicon with the standard effective masses $m_{\|}=0.91\;m_{0}$ and $m_{⊥}=0.19\;m_{0}$. Dashed lines for an averaged isotropic effective mass $m^{*}={\left(m_{\|}m_{⊥}^{2}\right)}^{1/3}=0.32\;m_{0}$.

**Figure A3:**
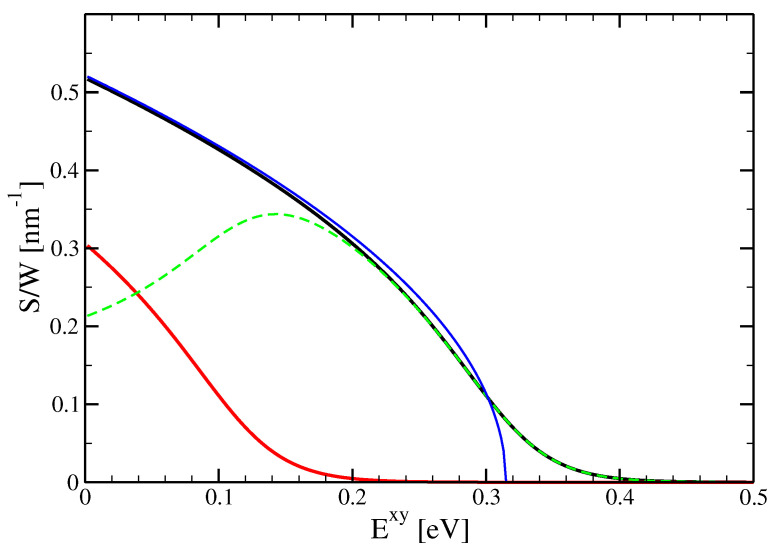
The supply functions $S(E^{xy}-\mu )$ (**black**) and $S(E^{xy}-\mu +eU_{D})$ (**red**) as well as $S\left(E^{xy}-\mu \right)-S\left(E^{xy}-\mu +eU_{D}\right)$ (**green dashed**) and $S(E^{xy}-\mu )$ in the limit T→0 (**blue**). As found in Appendix B, the chemical potential in the source is μ=0.315 eV. At T=300 K, we obtain $\lambda_{th}=7.58$ nm.

**Figure A5:**
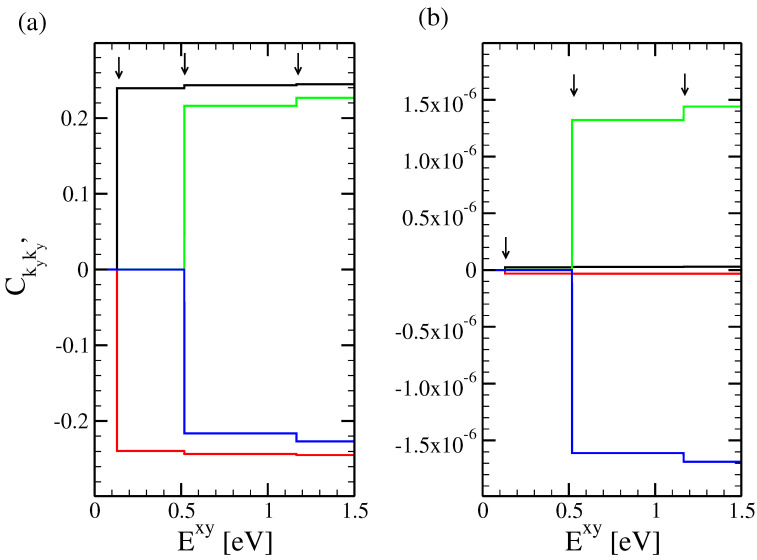
The wave function overlap $C_{k_{y}k_{y}^{\prime }}\left(E^{xy}\right)$ for the diagonal elements $k_{y}=k_{y}^{\prime }=1$ (**black**) and $k_{y}=k_{y}^{\prime }=3$ (**green**) and for the off-diagonal elements $k_{y}=1$, $k_{y}^{\prime }=2$ (**red**) as well as $k_{y}=3$, $k_{y}^{\prime }=4$ (**blue**). (**a**) $V_{g}=0$ and (**b**) $V_{g}=-0.1$. The arrows mark the position of the steps $E_{1}^{⊥y}$, $E_{2}^{⊥y}$ and $E_{3}^{⊥y}$. Further parameters: $V_{B}=2eV$ and $U_{D}=0$.

**Figure A6:**
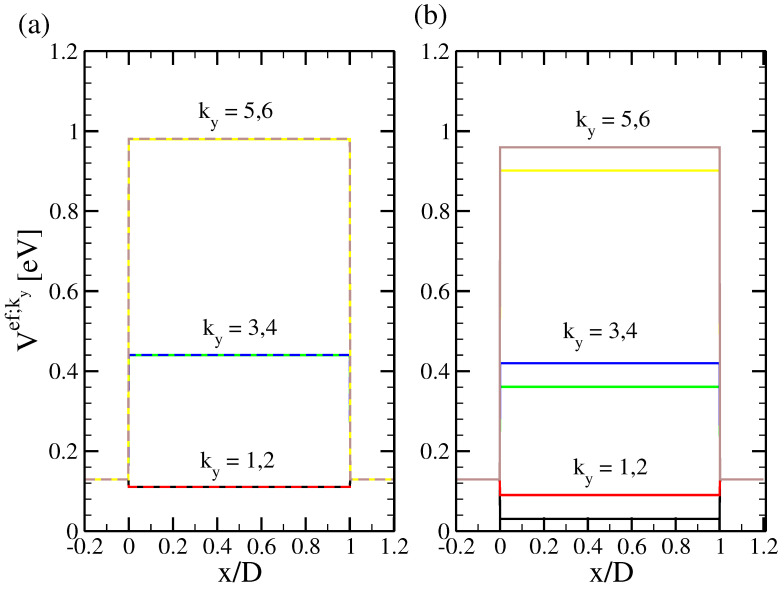
The effective potential at $V_{g}=0$ (**a**) and at $V_{g}=-0.1$ eV (**b**) for $k_{y}=1$ (**black**), 2 (**red**), 3 (**green**), 4 (**blue**), 5 (**yellow**), and 6 (**brown**). Further parameters: $V_{B}=2$ eV and $U_{D}=0$.

**Figure A7:**
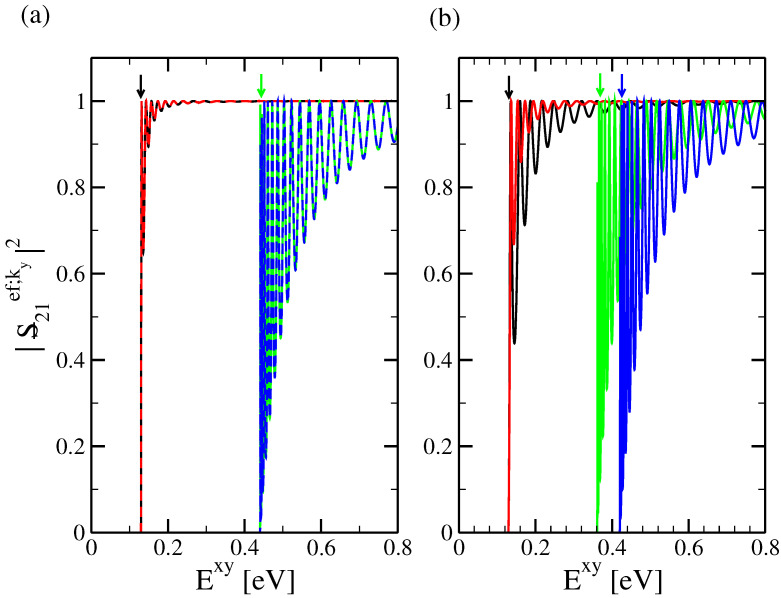
Square of the one-dimensional effective current transmission coefficient ${\tilde{S}}_{21}^{ef;k_{y}}$ in Equation (A17) at $V_{g}=0$ (**a**) and at $V_{g}=-0.1$ (**b**) for $k_{y}=1$ (**black**), 2 (**red**), 3 (**green**), 4 (**blue**). Further parameters: $V_{B}=2$ eV and $U_{D}=0$. Black arrows $E_{1}^{⊥y}$, green arrows $E_{3}^{Ty}$ and blue arrow $E_{4}^{Ty}$.

**Figure A8:**
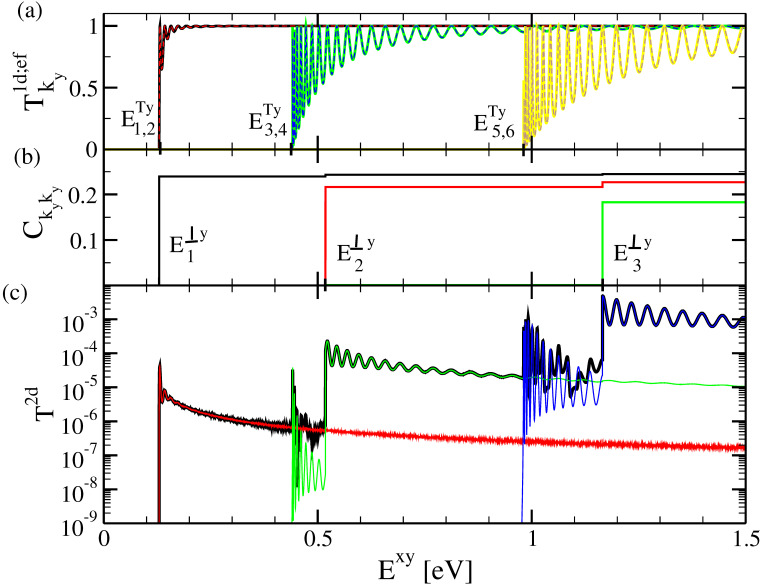
For $V_{B}=2eV$, $V_{g}=0$ and $U_{D}=0$: (**a**) The effective one-dimensional current transmission $T_{k_{y}}^{1d;ef}$ for $k_{y}=1,2$ (**solid black** and **red dashed**), $k_{y}=3,4$ (**solid green** and **dashed blue**), and $k_{y}=5,6$ (**solid yellow** and **dashed brown**). (**b**) The wave function overlap $C_{k_{y},k_{y}^{\prime }}$ for $k_{y}=k_{y}^{\prime }=1$ (**black**), $k_{y}=k_{y}^{\prime }=3$ (**red**) and $k_{y}=k_{y}^{\prime }=5$ (**green**). (**c**) The two-dimensional current transmission $T^{2d}$ calculated from (A14) with $k_{y}$ and $k_{y}^{\prime }$ in the entire range (fat, black), restricted to one and two (thin, red) to three and four (thin, green) and to five and six (thin, blue).

## Data Availability

The original contributions presented in the study are included in the article, further inquiries can be directed to the corresponding author.

## Abbreviations

The following abbreviations are used in this manuscript:

2CTFET	two-channel tunneling field-effect transistor
2D	two-dimensional
DELTT	double electron layer tunneling transistors
EAPS	effective approximation for planar systems
FET	field-effect transistor
MOSFET	metal oxide semiconductor field-effect transistor
SOI	silicon-on-insulator

## Appendix A. Transverse Modes in the Contacts

Inserting in the 2D Schrödinger Equation (5) in the source/drain contact the constant potential $V\left(\vec{r}\in \Omega_{s}\right)=-eU_{s}$ where s=1,2 is the contact index and $U_{1}=0$ as well as $U_{2}=U_{D}$

(A1) $$\psi \left(x,y\right)=\Phi_{sn_{y}}\left(y\right)\exp\left(ik_{sn_{y}}x\right)$$

one finds for the transverse modes in the y-direction $\Phi_{sn_{y}}$

(A2) $$\left[-\frac{\hbar^{2}}{2m^{*}}\frac{d^{2}}{dy^{2}}-E_{sn_{y}}^{⊥y}\right]\Phi_{sn_{y}}\left(y\right)=0.$$

Here, the transverse mode energy is given by

(A3) $$E_{sn_{y}}^{⊥y}=\frac{\hbar^{2}}{2m^{*}}{\left(\frac{n_{y}\pi }{D_{C}}\right)}^{2}$$

and the wavenumber is given by

(A4) $$k_{sn_{y}}=\frac{\sqrt{E^{xy}+eU_{s}-E_{sn_{y}}^{⊥y}}}{\hbar }.$$

We plot in Figure A1 the transverse modes in the source

(A5) $$\Phi_{1n_{y}}\left(y\right)=\Theta \left[y-\left(D_{B}+D_{C}\right)\right]\sqrt{\frac{2}{D_{C}}}\sin\left[\frac{n_{y}\pi }{D_{C}}\left(y-D_{B}-D_{C}\right)\right]$$

and in the drain

(A6) $$\Phi_{2n_{y}}\left(y\right)=\Theta \left(D_{C}-y\right)\sqrt{\frac{2}{D_{C}}}\sin\left(\frac{n_{y}\pi }{D_{C}}y\right).$$

The transverse mode energies in the two contacts are identical, $E_{sn_{y}}^{⊥y}=E_{n_{y}}^{⊥y}$ and are given in the following table.

**Table A1:** Transverse mode energies for $D_{C}=3$ nm.

$n_{y}$	1	2	3	4
$E_{n_{y}}^{⊥y}$ in eV	0.1295	0.5180	1.1654	2.0718

## Appendix B. Chemical Potential in the Source/Drain Contacts

To calculate the chemical potential in the source contact, we adopt the approach in [23]: The source/drain contacts are identical cubic flat volumes of length L in the x-direction, depth $D_{C}$ in the y-direction and *W* in the z-direction. Imposing fixed boundary conditions in all three space dimensions, we write for the number of free charge carriers

(A7) $$N=2\sum_{\alpha }\sum_{n_{x}n_{z}n_{x}=1}^{\infty }f\left(\frac{\hbar^{2}}{2m_{x}^{\alpha }}\frac{n_{x}^{2}\pi^{2}}{L^{2}}+\frac{\hbar^{2}}{2m_{y}^{\alpha }}\frac{n_{y}^{2}\pi^{2}}{D_{C}^{2}}+\frac{\hbar^{2}}{2m_{z}^{\alpha }}\frac{n_{z}^{2}\pi^{2}}{W^{2}}-\mu \right).$$

Here, $f={\left\{\exp\left[x/\left(k_{B}T\right)\right]+1\right\}}^{-1}$ is the Fermi–Dirac distribution; in silicon, there are six constant energy ellipsoids α=1…6 with effective masses $m_{x}^{\alpha },m_{y}^{\alpha },m_{z}^{\alpha }$ and we include spin degeneracy with a factor of two. In the next step, the free carrier density *n* is introduced with $N=nLD_{C}W=N_{D}LD_{C}W$ assuming full ionization of the donors so that $n=N_{D}$ with the donor concentration $N_{D}$ equal in source and drain. In the limit L→∞ and W→∞, one obtains the relation

(A8) $$1=\frac{2}{N_{D}D_{C}}\sum_{\alpha }\sum_{n_{y}}\frac{1}{\pi^{2}}\int_{0}^{\infty }\int_{0}^{\infty }dk_{x}dk_{z}\frac{1}{\exp\left[\frac{1}{k_{B}T}\left(\frac{\hbar^{2}}{2m_{x}^{\alpha }}k_{x}^{2}+\frac{\hbar^{2}}{2m_{z}^{\alpha }}k_{z}^{2}+\frac{\hbar^{2}}{2m_{y}^{\alpha }}\frac{n_{y}^{2}\pi^{2}}{D_{C}^{2}}-\mu \right)\right]+1}$$

setting $\sum_{n_{x}}..\to \left(L/\pi \right)\int dk_{x}\ldots$ and $\sum_{n_{z}}..\to \left(W/\pi \right)\int dk_{z}\ldots$. After some further technical steps which are described in Ref. [23], this equation is solved numerically to find μ as depicted in Figure A2. The chemical potential in the drain is lowered by the applied drain voltage to $\mu -eU_{D}$.

## Appendix C. Supply Function

We adopt results from Section 7 of [19] and define the supply function as

(A9) $$S\left(\alpha \right)=\sum_{n_{z}}f\left[\alpha +\frac{\hbar^{2}}{2m^{*}}{\left(\frac{n_{z}\pi }{W}\right)}^{2}\right].$$

In the limit W→∞, we can write

(A10) $$S\left(\alpha \right)=\frac{W}{\lambda_{th}}F_{-1/2}\left(-\frac{\alpha }{k_{B}T}\right)$$

where $\lambda_{th}={\left[2\pi \hbar^{2}/\left(m^{*}k_{B}T\right)\right]}^{1/2}$ is the thermal de Broglie wavelength and the Fermi–Dirac-Integral is given by

(A11) $$F_{-1/2}\left(x\right)=\frac{1}{\sqrt{\pi }}\int_{0}^{\infty }dyy^{-1/2}\frac{1}{1+e^{y-x}}.$$

In Figure A3, we plot the supply function at room temperature together with the zero-temperature limit

(A12) $$S\left(\alpha \right)\to W\sqrt{\frac{2m^{*}}{\pi^{2}\hbar^{2}}}\Theta \left(-\alpha \right){\left|\alpha \right|}^{1/2}.$$

As expected, the supply functions restrict the energy integral in the Landauer–Büttiker Formula (6) to values below the chemical potential including also a small thermal broadening in the order of the thermal energy.

## Appendix D. Transverse Modes in the Device Kernel

In the separable potential (1), one can define transverse modes as the solutions of the the Schrödinger equation

(A13) $$\left[-\frac{\hbar^{2}}{2m^{*}}\frac{d^{2}}{dy^{2}}+V_{T}\left(y\right)-E_{k_{y}}^{Ty}\right]\zeta_{k_{y}}\left(y\right)=0.$$

The functions $\zeta_{k_{y}}$ are the transverse modes in the *y*-direction in the kernel (see Equation (61) of [19]) and the $E_{k_{y}}^{Ty}$ are their energies. As described in Appendix E, they enter $T^{2d}$ via the wave function overlap $C_{k_{y}k_{y}^{\prime }}\left(E^{xy}\right)$.

With the aid of the transverse modes in the kernel, one can formulate a first intuitive understanding of the resonant transverse tunneling transport: in Figure 8, we plot the two lowest transverse modes with $k_{y}=1,2$. In contrast to the higher energetic transverse modes, we have $E_{k_{y}=1,2}^{⊥}<\mu$ and these states therefore dominate the drain current due to the high-energy decay cut-off in the supply function shown in Figure A3. In the symmetrical case at $V_{g}=0$, the top and the bottom quantum well are in resonance. Then, the transverse modes in the kernel are localized in both wells simultaneously and current can flow between the two wells (see Figure 8a). A charge carrier that is injected from the source in the bottom quantum well can exit in the top quantum well to create a drain current. In the asymmetrical system at $V_{g}=0.1$ V, the top and the bottom quantum well are out of resonance. Then the transverse modes in the kernel are localized either exclusively in the bottom quantum well or exclusively in the top quantum well and no charge can transit between the two wells (see Figure 8b). A charge carrier injected from the source in the bottom quantum well is totally reflected and no drain current results. This is the strongly idealized basic operating principle of the 2CTFET which is substantially modified by the coupling of the two modes $k_{y}=1,2$ as described in Appendix E.1.

For the parameter values in Figure A4, we find the following transverse mode energies.

In the symmetrical potential at $U_{g}=0$, the lowest transverse modes come in pairs of nearly degenerate states, one symmetrical state (i.e., $k_{y}=1$) and one asymmetrical state (i.e., $k_{y}=2$). Therefore, the dominant transverse modes with $k_{y}=1$ and $k_{y}=2$ strongly interact when the current is driven through the transistor as a perturbation. This is seen from the wave function overlap $C_{12}$ which is plotted in Figure A6 and discussed in detail in Appendix E.1.

**Table A2:** Transverse mode energies for the parameters in Figure A4.

$k_{y}$	1	2	3	4	5	6
$E_{k_{y}}^{Ty}$ in eV for $V_{g}=0$	0.1105	0.1106	0.4401	0.4402	0.9800	0.9811
$E_{k_{y}}^{Ty}$ in eV for $V_{g}=-0.1V$	0.0307	0.0901	0.3606	0.4198	0.9014	0.9597

## Appendix E. Two-Dimensional Current Transmission in EAPS

In the effective approximation for planar systems, the two-dimensional current transmission is given by

(A14) $$T^{2d}\left(E^{xy}\right)=\sum_{k_{y},k_{y}^{\prime }}C_{k_{y}k_{y}^{\prime }}\left(E^{xy}\right){\tilde{S}}_{21}^{ef;k_{y}}{\left({\tilde{S}}_{21}^{ef;k_{y}^{\prime }}\right)}^{*}$$

(See Section 7 of [19]). Here, $C_{k_{y}k_{y}^{\prime }}$ is the wave function overlap which is discussed in detail in Appendix E.1 and ${\tilde{S}}_{21}^{ef;k_{y}}$ is the one-dimensional effective current S-matrix discussed in Appendix E.2. In Appendix E.3, the function $T^{2d}\left(E^{xy}\right)$ is evaluated.

### Appendix E.1. Wave Function Overlap

In Figure A5, we plot the wave function overlap entering Equation (A14) which is given by Equation (68) of [19]

(A15) $$\begin{array}{ccc} C_{k_{y}k_{y}^{\prime }}\left(E^{xy}\right) & = & \sum_{n_{y}n_{y}^{\prime }}^{\infty }{\bar{c}}_{k_{y}1n_{y}}{\bar{c}}_{k_{y}2n_{y}^{\prime }}{\bar{c}}_{k_{y}^{\prime }1n_{y}}{\bar{c}}_{k_{y}^{\prime }2n_{y}^{\prime }}\Theta \left[E^{xy}-E_{1n_{y}}^{⊥y}\right]\Theta \left[E^{xy}-E_{2n_{y}^{\prime }}^{⊥y}+eU_{D}\right]\\ & = & \sum_{n_{y}n_{y}^{\prime }}^{\infty }{\bar{c}}_{k_{y}1n_{y}}{\bar{c}}_{k_{y}2n_{y}^{\prime }}{\bar{c}}_{k_{y}^{\prime }1n_{y}}{\bar{c}}_{k_{y}^{\prime }2n_{y}^{\prime }}\Theta \left[E^{xy}-E_{n_{y}}^{⊥y}\right]\Theta \left[E^{xy}-E_{n_{y}^{\prime }}^{⊥y}+eU_{D}\right] \end{array}$$

with the overlap factors in the y-direction

(A16) $${\bar{c}}_{k_{y}sn_{y}}=\int_{0}^{D}dy\Phi_{sn_{y}}\left(y\right)\zeta_{k_{y}}\left(y\right).$$

Here, the Θ-functions in Equation (A15) describe the active transverse modes in the contacts and the overlap factors describe the quality of the coupling between the active transverse modes in the contacts to the transverse modes in the kernel.

It is seen that the wave function overlap decreases strongly with $|V_{g}|$ in the range of the resonant tunneling peak starting from its maximum at $V_{g}=0$ and going over to the local minimum around $|V_{g}|∼0.1$ eV. This strong change in the wave function overlap causes the resonant tunneling peak in the transfer characteristics shown in Figure 2. In the relevant energy range $0<E^{xy}<0.5$ eV with a non-vanishing supply function (Figure A3), only the positive diagonal elements $C_{11}$ and $C_{22}$ and the negative off-diagonal elements $C_{21}$ and $C_{12}$ are essential. The diagonal elements represent the independent contributions of the transverse modes in the kernel and the off-diagonal elements their coupling. In the center of the resonant tunneling peak ($V_{g}=0$), the absolute value of the diagonal elements and of the off-diagonal elements are nearly the same, having opposite signs. Therefore, the off-diagonal elements nearly cancel out the diagonal ones, indicating strong destructive coupling of the two modes with $k_{y}=1$ and $k_{y}=2$. These two modes are shown in Figure 4, but we can generalize the destructive coupling to all pairs of nearly degenerate modes at $V_{g}=0$ which appear in the upper row of Table A2.

### Appendix E.2. One-Dimensional Current Transmission

Following Section D of [19], we can write

(A17) $${\bar{S}}_{21}^{ef;k_{y}}=\sqrt{\frac{k_{2}^{ef}}{k_{1}^{ef}}}t^{ef;k_{y}}$$

where the effective transmission coefficients $t^{ef;k_{y}}$ are calculated from the scattering solutions of a one-dimensional effective Schrödinger equation

(A18) $$\left[-\frac{\hbar^{2}}{2m^{*}}\frac{d^{2}}{dx^{2}}+V^{ef;k_{y}}\left(x\right)-E^{xy}\right]\psi^{ef;k_{y}}\left(x\right)=0$$

with the effective scattering potential

(A19) $$V^{ef;k_{y}}\left(x\right)=\left\{\begin{array}{cc} E_{1}^{⊥y} & \mathrm{for}\;x<0\\ E_{k_{y}}^{Ty}+V_{L}\left(x\right)\; & \mathrm{for}\;0\leq x\leq L\\ E_{1}^{⊥y}-eU_{D} & \mathrm{for}\;x>L \end{array}\right.$$

(see Figure A6). For $k_{y}\geq 3$, the effective potential has the character of a tunneling barrier while for $k_{y}=1,2$ the effective potential has the character of a quantum well. The latter case is caused by the fact that for $D_{C}=D_{W}$ the possibility of resonant tunneling in the device kernel leads to $E_{1}^{⊥y}>E_{1,2}^{Ty}$.

The asymptotics of the source incident scattering states of the scattering problem associated with (A18) are given by

(A20) $$\psi^{ef;k_{y}}\left(x<0\right)=\Theta \left(E^{xy}-E_{1}^{⊥y}\right)\left(e^{ik_{1}^{ef}x}+r^{ef;k_{y}}e^{-ik_{1}^{ef}x}\right),$$

and

(A21) $$\psi^{ef;k_{y}}\left(x\geq L\right)=\Theta \left(E-E_{1}^{⊥y}\right)t^{ef;k_{y}}e^{ik_{2}^{ef}\left(x-L\right)}$$

where

(A22) $$k_{s}^{ef}\left(E^{xy}\right)=\sqrt{\frac{2m^{*}}{\hbar^{2}}}\left[E-E_{1}^{⊥y}+eU_{s}\right].$$

As can be taken from Figure A7, the one-dimensional effective current transmission $|{\bar{S}}_{21}^{ef;k_{y}}{|}^{2}$ shows for $k_{y}\geq 3$ the well-known turnover from zero to unity around the maxima of the effective potential which are given by $E_{k_{y}}^{Ty}$. For $k_{y}=1,2$ the turnover to unity current transmission occurs at $E_{1}^{⊥y}$ because of the theta-function in (A21). At all $k_{y}$, one finds the superimposed well-known Fabry–Perot oscillations. Because $E_{3,4}^{Ty}∼0.4eV$, the cut-off in the supply function around μ=0.351 causes the main contribution to the drain current to come from $k_{y}=1,2$.

### Appendix E.3. Numerical Evaluation of the Two-Dimensional Current Transmission

We find from Equation (A14) the two-dimensional current transmission $T^{2d}$ as plotted in Figure A8c.

Because of the associated steps in the wave function overlap shown in Figure A8b, the two-dimensional current transmission takes the form of a series of steps at $E_{n_{y}}^{⊥y}$. Therefore, the steps and the following ‘plateaus’ can be numbered with $n_{y}$. The steps result from the addition of active transverse modes in the contacts when $E^{xy}$ reaches $E_{n_{y}}^{⊥y}$. Within each plateau, there exists a transition energy which is given by a certain $E_{k_{y}}^{Ty}$: For lower energies, one observes regular Fabry–Perot oscillations (‘Fabry–Perot regime’), whereas for higher energies the current transmission develops an erratic behavior (‘erratic regime’). In the Fabry–Perot regime, $T^{2d}$ is dominated by the two associated transverse modes in the kernel which match with the incident transverse mode $n_{y}$ in the contacts (‘matching modes’); i.e., for $n_{y}=1$ the matching modes are $k_{y}=1,2$, for $n_{y}=2$ they are $k_{y}=3,4$, for $n_{y}=3$ they are $k_{y}=5,6$ and so on. In the previous section, we mentioned the strong destructive coupling of these pairs of matching modes, which leads to relatively small values of $T^{2d}$. For higher energies, at the transition to the erratic regime of a given $n_{y}$-plateau, transverse modes in the kernel with higher $k_{y}$ than the matching modes are mixed in (‘mixed in modes’). For example, for $n_{y}=1$ the mixed in modes are with $k_{y}=3,4$, for $n_{y}=2$ mixed-in modes are with $k_{y}=5,6$ and so on. The transition energies to the erratic regime are therefore the energies $E_{k_{y}}^{Ty}$ of these mixed-in modes. The erratic behavior stems from the interference between the comparable contributions of the matching modes and the mixed-in modes.

In Figure A9, we compare $T^{2d}$ calculated in the EAPS with the result of the complete R-matrix method and with the results of Kwant [25]. In all three approaches, there are three steps in the current transmission in the considered energy range. In each step, there is the described transition from a Fabry–Perot regime to an erratic regime. The step energies $E_{n_{y}}^{⊥y}$ and the transition energies $E_{k_{y}}^{Ty}$ of the EAPS and the fully numerical evaluations agree well. The Fabry–Perot oscillations are much weaker in the EAPS, their period agrees with that in the fully numerical evaluations but there can be a phase shift which probably is caused by the approximation described in Figure 1d,e. If one averages out the Fabry–Perot oscillations on the logarithmic scale, the EAPS seems to give the correct overall behavior in an $n_{y}$-plateau, i.e., a general decrease in the current transmission with increasing energies which becomes weaker with increasing $n_{y}$. However, in absolute numbers the EAPS underestimates the current transmission considerably.
